# III-Nitride grating grown on freestanding HfO_2 _gratings

**DOI:** 10.1186/1556-276X-6-497

**Published:** 2011-08-18

**Authors:** Yongjin Wang, Tong Wu, Fangren Hu, Yoshiaki Kanamori, Hongbo Zhu, Kazuhiro Hane

**Affiliations:** 1 Institute of Communication Technology, Nanjing University of Posts and Telecommunications, Nanjing, Jiang-Su 210003, People's Republic of China; 2 Department of Nanomechanics, Tohoku University, Sendai 980-8579, Japan

**Keywords:** InGaN/GaN QWs, fast atom beam etching, molecular beam epitaxy

## Abstract

We report here the epitaxial growth of III-nitride material on freestanding HfO_2 _gratings by molecular beam epitaxy. Freestanding HfO_2 _gratings are fabricated by combining film evaporation, electron beam lithography, and fast atom beam etching of an HfO_2 _film by a front-side silicon process. The 60-μm long HfO_2 _grating beam can sustain the stress change during the epitaxial growth of a III-nitride material. Grating structures locally change the growth condition and vary indium composition in the InGaN/GaN quantum wells and thus, the photoluminescence spectra of epitaxial III-nitride grating are tuned. Guided mode resonances are experimentally demonstrated in fabricated III-nitride gratings, opening the possibility to achieve the interaction between the excited light and the grating structure through guided mode resonance.

**PACS: **78.55.Cr; 81.65.Cf; 81.15.Hi.

## Introduction

Freestanding III-nitride structures can take advantage of the large refractive index contrast between III-nitride and air [1-7]. In such structure, the excited light has a potential to interact with freestanding structure through guided modes. Among the approaches towards creating suspended III-nitride structures, growth of III-nitride on freestanding structured template is an emerging technology. During growth process, nanoscale structures locally change the growth conditions and thus, the selective growth can be achieved to generate epitaxial III-nitride structures with smooth facets [8-11]. Meanwhile, freestanding III-nitride structures are formed by growth method and free of the etching damage. Moreover, the as-grown III-nitride structures can provide a natural optical cavity to support guided mode resonances, opening the possibility to achieve the interaction between the excited light and the epitaxial structures.

From the growth point of view, small material lattice mismatch between HfN and GaN crystals makes HfN film a superior buffer layer for the growth of GaN [12,13]. During molecular beam epitaxy (MBE) growth, HfN surface can be formed by nitrifying HfO_2 _substrate. Hence, structured HfO_2 _film can be used as a template for growing III-nitride materials [14]. On the other hand, HfO_2 _film is an excellent optical material with high laser damage threshold, thermal, and chemical stability [15-17]. Recently, we have fabricated freestanding HfO_2 _gratings and experimentally demonstrated their guided mode resonances [18]. It is of great interest to implement the growth of III-nitride materials on freestanding HfO_2 _gratings.

Here, we demonstrate the freestanding III-nitride gratings grown on suspended HfO_2 _gratings. The epitaxial growth of InGaN/GaN quantum wells (QWs) are performed on freestanding HfO_2 _gratings by MBE technique. The optical performances of the resultant epitaxial structures are characterized in photoluminescence (PL) and reflectance measurements.

### Fabrication

**Figure 1:**
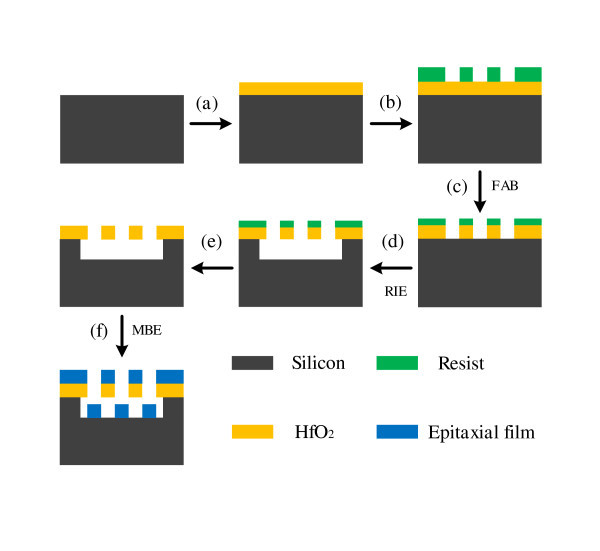
**Schematic process of III-nitride grating grown on freestanding HfO_2 _grating by MBE**.

The whole fabrication process is schematically illustrated in Figure 1. Freestanding HfO_2 _gratings are fabricated by a combination of film evaporation, electron beam lithography, fast atom beam (FAB) etching of HfO_2 _film with a front-side silicon process [18]. Subsequently, the epitaxial growth of III-nitride material by MBE is conducted on the suspended HfO_2 _gratings. After nitrifying HfO_2 _substrate at the temperature of 680°C for 20 min, a 150-nm thick buffer layer is deposited at a temperature of 680°C, and a 450-nm high-temperature GaN layer is then grown at the temperature of 750°C. The six-pair 3-nm InGaN/12-nm GaN MQWs is subsequently deposited at a temperature of 660°C. Finally, a 20-nm GaN layer is grown at a temperature of 660°C.

### Experimental results and discussion

**Figure 2:**
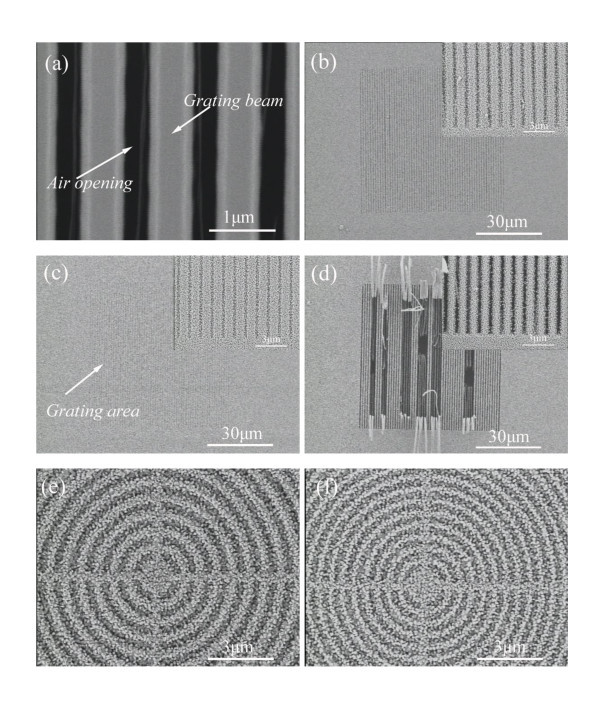
**SEM images of a freestanding HfO_2 _grating template**. **(a) **SEM image of freestanding HfO_2 _grating template; **(b) **epitaxial III-nitride grating with air opening of 400 nm, and the inset is the zoom-in image; **(c) **epitaxial III-nitride grating with an air opening of 200 nm, and the inset is the zoom-in image; **(d) **epitaxial III-nitride grating with an air opening of 600 nm, and the inset is the zoom-in image; **(e) **700-nm period epitaxial circular III-nitride grating; and **(f) **500-nm period epitaxial circular III-nitride grating.

Figure 2a illustrates scanning electron microscope (SEM) images of freestanding HfO_2 _grating template with a grating period of 1,020 nm. One period grating is comprised of HfO_2 _grating beam and air opening. Actually, a suspended HfO_2 _grating beam functions as a template for the epitaxial growth of III-nitride materials. Since the original HfO_2 _grating beam has a trapezoidal profile, we use the designed air opening to describe the epitaxial III-nitride grating for simplicity. Figure 2b shows the 60-period epitaxial grating grown on 1,020 nm period suspended HfO_2 _grating with the air opening of 400 nm, and the inset is the zoom-in image of freestanding III-nitride grating. The 60-μm long suspended HfO_2 _grating beam can guarantee sufficient stiffness for MBE growth. III-Nitride nanocolumns are grown on the top surface and the sidewall of the grating beam. The flat III-nitride grating surface can be achieved by improving the growth condition. Decreasing the original air opening to 200 nm, the resultant grating opening illustrated in the inset of Figure 2c is easily filled with III-nitride nanocolumns, and the epitaxial III-nitride grating is similar to that grown on unpatterned area, as shown in Figure 2c. Increasing the original air opening to 600 nm, the epitaxial grating beams shown in Figure 2d are in the tendency of being deflected and fragile. Figure 2e,f show the epitaxial circular gratings with grating period of 700 and 500 nm, respectively. It can be clearly seen that the grating openings are easily filled with III-nitride nanocolumns as the grating period decreases, especially for small period grating.

**Figure 3:**
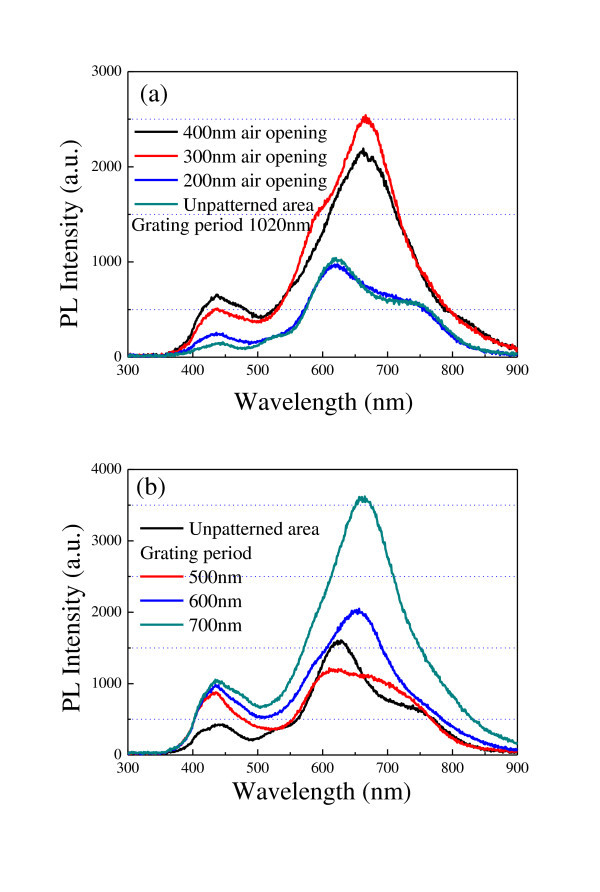
**PL spectra of epitaxial gratings with various air openings**. **(a) **PL spectra of 1,020-nm period epitaxial III-nitride grating versus air opening. **(b) **PL spectra of epitaxial III-nitride grating versus grating periods.

The emission properties of the resultant epitaxial structures are characterized using a micro-PL system at room temperature. The excitation source is a continuous wave 325 nm He-Cd laser source, and the pump light is focused on the sample through a UV-compatible objective lens (×20 and numerical aperture, 0.36). The emitted light is collected by the same objective lens and measured using a multichannel analyzer system (Hamamatsu C10027). Figure 3a shows the PL spectra of 1,020-nm period epitaxial gratings with various air openings. Regarding the excitation of InGaN/GaN QWs grown on an unpatterned area, two distinct PL peaks are observed around 441 and 620.5 nm, and a clear broad shoulder is found at approximately 748.7 nm in the PL spectra. The emission from epitaxial materials is dependent on indium content in the InGaN quantum well [19]. Variations in growth temperature leads to indium composition fluctuations and eventually results in the broad emission spectra [20-22]. Compared with unpatterned substrates, grating structures locally change the epitaxial growth conditions, giving an influence on indium composition in the epitaxial gratings. It can be seen that the tuning of the PL spectra is much more obvious as the original air opening increases. As to the grating with a designed air opening of 400 nm, only two PL peaks are observed around 437.9 and 662.6 nm, respectively. The PL intensity is also improved for epitaxial grating structures due to their freestanding characteristics. Figure 3b illustrates the PL spectra of circular epitaxial gratings versus grating period. As the grating period decreases, the measured PL spectra tend to be similar to those of unpatterned area, except a distinct increase in PL intensity of approximately a 434-nm peak.

**Figure 4:**
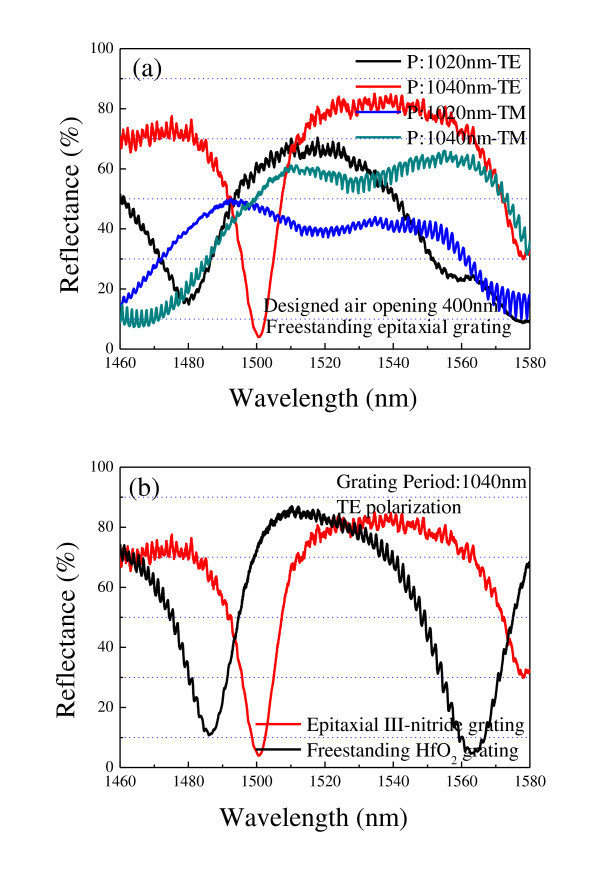
**Illustration of the reflection dip**. **(a) **Reflectance spectra of epitaxial III-nitride grating. **(b) **Comparison of reflectance spectra between HfO_2 _grating and resultant III-nitride grating.

Since the epitaxial gratings are freely suspended with air as the low refractive index material on the top and bottom, the freestanding epitaxial gratings can serve as an optical cavity to support guided mode resonances, which are characterized in reflectance measurement with the tunable laser operating in the range of 1,460 nm to approximately 1,580 nm. The optical responses of the freestanding epitaxial gratings are dependent on the polarization due to their one-dimensional configuration. The reflectance spectra versus polarization are obtained by rotating the sample with an angle of 90° with respect to the initial measurement. Under transverse electric (TE) polarization (TE is polarized in the plane of the grating and parallel to the grating lines), one sharp reflection dip approximately 4% is clearly observed at 1,500.5 nm for 1,040 nm period epitaxial grating. Measured reflectances over 70% are with a broad stopband in the range of 1,512.8 nm to approximately 1,563.5 nm. The reflection band shifts, and the shape changes under transverse magnetic polarization, which is perpendicular to the grating lines. As the grating period decreases from 1,040 to 1,020, the broad reflection band exhibits a distinct blue shift, indicating that the guided mode resonances shift proportionally to the grating period. Compared with the original HfO_2 _gratings, the effective index of the freestanding grating is increased after the epitaxial growth of III-nitride materials, resulting in the red shifting of reflectance spectra and the broadening of the reflection band. The reflection dip illustrated in Figure 4b has a red shift of approximately 15 nm, and the reflection band widens about 10 nm. Period-, polarization-, and refractive index-dependent resonances endow freestanding III-nitride gratings the capacities to be used for resonant optical devices. Since InGaN/GaN QWs are incorporated into the grating structures, another potential application of resonant III-nitride grating is developed for the resonant emission, where the excited light from QWs is coupled to one mode of the grating waveguide due to the match between emission wavelength and resonant wavelength.

## Conclusions

The epitaxial growth of III-nitride material is performed on the suspended HfO_2 _grating. The 60-μm long freestanding HfO_2 _grating beam can sustain the stress change during MBE growth. The PL spectra and reflectance of epitaxial III-nitride gratings are experimentally characterized. Epitaxial III-nitride grating can function as an optical cavity to support resonance mode, which is demonstrated and compared to the resonances of original HfO_2 _grating. These results indicate that resonant III-nitride gratings are promising for the development of resonant optical devices and the realization of the resonant emission. This work also opens the possibility for fabricating novel III-nitride optic devices by a combination of freestanding HfO_2 _nanostructures with epitaxial growth of III-nitride materials.

## Competing interests

The authors declare that they have no competing interests.

## Authors' contributions

YW carried out the device design and fabrication, performed the optical measurements, and drafted the manuscript. TW carried out HfO_2 _film evaporation. FH conducted the epitaxial growth of III-nitride. YK participated in its design and optical characterization. HZ participated in the draft of the manuscript and coordination. KH conceived of the study, and participated in its design and coordination. All authors read and approved the final manuscript.

## References

[B1] Rosenberg A Carter M Casey J Kim M Holm R Henry R Eddy C Shamamian V Bussmann K Shi S Prather D Guided resonances in asymmetrical GaN photonic crystal slabs observed in the visible spectrum Opt Express 2005 13 6564 6571 10.1364/OPEX.13.006564 19498672

[B2] Choi HW Hui KN Lai PT Chen P Zhang XH Tripathy S Teng JH Chua SJ Lasing in GaN microdisks pivoted on Si Appl Phys Lett 2006 89 211101 10.1063/1.2392673

[B3] Meier C Hennessy K Haberer ED Sharma R Choi YS McGroddy K Keller S DenBaars SP Nakamura S Hu EL Visible resonant modes in GaN-based photonic crystal membrane cavities Appl Phys Lett 2006 88 031111 10.1063/1.2166680

[B4] Rosenberg A Bussmann K Kim M Carter MW Mastro MA Holm RT Henry RL Caldwell JD Eddy CR Jr Fabrication of GaN suspended photonic crystal slabs and resonant nanocavities on Si(111) J Vac Sci Technol B 2007 25 3 721 10.1116/1.2723750

[B5] Arita M Ishida S Kako S Iwamoto S Arakawa Y AlN air-bridge photonic crystal nanocavities demonstrating high quality factor Appl Phys Lett 2007 91 051106 10.1063/1.2757596

[B6] Cimalla V Pezoldt J Ambacher O Group III nitride and SiC based MEMS and NEMS: materials properties, technology and applications J Phys D: Appl Phys 2007 40 6386 10.1088/0022-3727/40/20/S19

[B7] Matsubara H Yoshimoto S Saito H Yue JL Tanaka Y Noda S GaN Photonic-crystal surface-emitting laser at blue-violet wavelengths Science 2008 319 445 447 10.1126/science.1150413 18096768

[B8] Kikuchi A Kawai M Tada M Kishino K InGaN/GaN multiple quantum disk nanocolumn light-emitting diodes grown on (111) Si substrate Jpn J Appl Phys 2004 43 L1524 10.1143/JJAP.43.L1524

[B9] Kishino K Sekiguchi H Kikuchi A Improved Ti-mask selective-area growth (SAG) by rf-plasma-assisted molecular beam epitaxy demonstrating extremely uniform GaN nanocolumn arrays J Cryst Growth 2009 311 2063 2068 10.1016/j.jcrysgro.2008.11.056

[B10] Wang YJ Hu FR Hane K Patterned growth of InGaN/GaN quantum wells on freestanding GaN grating by molecular beam epitaxy Nanoscale Res Lett 2011 6 117 10.1186/1556-276X-6-117 21711618 PMC3211162

[B11] Wang YJ Hu FR Hane K Lateral epitaxial overgrowth of GaN on patterned GaN-on-silicon substrate by molecular beam epitaxy Semicond Sci Technol 2011 26 045015 10.1088/0268-1242/26/4/045015

[B12] Armitage R Yang Q Feick H Gebauer J Weber ER Shinkai S Sasaki K Lattice-matched HfN buffer layers for epitaxy of GaN on Si Appl Phys Lett 2002 81 1450 1452 10.1063/1.1501447

[B13] Xu X Armitage R Shinkai S Sasaki K Kisielowski C Weber ER Epitaxial condition and polarity in GaN grown on a HfN-buffered Si(111) wafer Appl Phys Lett 2005 86 182104 10.1063/1.1923192

[B14] Sameshima H Wakui M Hu FR Hane K A freestanding GaN/HfO_2 _membrane grown by molecular beam epitaxy for GaN-Si hybrid MEMS IEEE J Sel Top Quantum Electron 2009 15 1332 1337

[B15] Priambodo PS Maldonado TA Magnusson R Fabrication and characterization of high-quality waveguide-mode resonant optical filters Appl Phys Lett 2003 83 3248 10.1063/1.1618930

[B16] Wang Y Lin Z Cheng X Xiao H Zhang F Zou S Study of HfO2 thin films prepared by electron beam evaporation Apple Surf Sci 228 93

[B17] Chaganti K Salakhutdinov I Avrutsky I Auner G Mansfield J Sub-micron grating fabrication on hafnium oxide thin-film waveguides with focused ion-beam milling Opt Express 2006 14 1505 10.1364/OE.14.001505 19503475

[B18] Wang YJ Wu T Kanamori Y Hane K Freestanding HfO_2 _grating fabricated by fast atom beam etching Nanoscale Res Lett 2011 6 367 10.1186/1556-276X-6-367 21711898 PMC3211457

[B19] Kuykendall T Ulrich P Aloni S Yang PD Complete composition tunability of InGaN nanowires using a combinatorial approach Nat Mater 2007 6 951 956 10.1038/nmat2037 17965718

[B20] Houdré R Stanley RP Ilegems M Vacuum-field Rabi splitting in the presence of inhomogeneous broadening: resolution of a homogeneous line width in an inhomogeneously broadened system Phys Rev A 1996 53 2711 2715 10.1103/PhysRevA.53.2711 9913184

[B21] Andreani LC Panzarini G Kavokin AV Vladimirova MR Effect of inhomogeneous broadening on optical properties of excitons in quantum wells Phys Rev B 1998 57 4670 10.1103/PhysRevB.57.4670

[B22] Christmann G Butteé R Carlin J-F Mosca M Grandjean N Feltin E Room temperature polariton luminescence from a GaN/AlGaN quantum well microcavity Appl Phys Lett 2006 89 071107 10.1063/1.2335404

